# The Role of Religion in Buffering the Impact of Stressful Life Events on Depressive Symptoms in Patients with Depressive Episodes or Adjustment Disorder

**DOI:** 10.3390/ijerph16071238

**Published:** 2019-04-08

**Authors:** Louisa Lorenz, Anne Doherty, Patricia Casey

**Affiliations:** 1 Department of Psychology, Division Psychopathology and Clinical Intervention, University of Zurich, 8057 Zurich, Switzerland; 2 Klinik im Hasel, Stationaere Therapie, 5728 Gontenschwil, Switzerland; 3 Liaison Psychiatry, University Hospital, H91 YR71 Galway, Ireland; Anne.Doherty5@hse.ie; 4 Mater Misericordiae University Hospital, Eccles St., D07 R2WY Dublin, Ireland; profcasey@esatclear.ie; 5 Department of Adult Psychiatry, University College Dublin, D07 R2WY Dublin, Ireland

**Keywords:** religiousness, organised religious practice, non-organised religious practice, intrinsic religiousness, social support

## Abstract

Most studies into the role of religiousness in relation to depression severity have mainly found an inverse relationship between greater religiousness and lower levels of depressive symptoms. There is reason to assume that religiousness has a buffering effect on the relationship between stressful life events and depressive symptoms. The aim of this study was to investigate the role of religiousness in moderating the impact of stressors on depressive symptoms. *n* = 348 patients with either a depressive episode or adjustment disorder were assessed at referral to the liaison psychiatry services in three Dublin hospitals and *n* = 132 patients were followed up six months later. We assessed depressive symptoms, life events, social support, and religiosity, and used hierarchical and multiple linear regression for data analysis. The interaction of organised religious activity and the amount of life events was significant (*β* = −0.19, *p* = 0.001) in the cross-sectional prediction of depressive symptoms while non-organised religious activity (*β* = −0.23, *p* = 0.001) and intrinsic religiousness (*β* = −0.15, *p* = 0.033) interacted significantly with life events in the longitudinal analysis. This study demonstrated that various dimensions of religiousness buffered the impact of life events on outcome.

## 1. Introduction

Stressful life events can act as triggers to many psychiatric disorders, in particular depressive episodes [1], post-traumatic stress disorder (PTSD) [2], and adjustment disorder (AD) [2]. Not everybody experiencing a stressful life event will experience a psychiatric disorder and among those who do, the severity may vary. With the growth of interest in resilience, attention is increasingly focused on its role in assisting people in dealing positively with stressors or alternatively in buffering the impact of such stressors on subsequent mental health. The most researched aspect is the role of social support [3], but the role of religiousness/spirituality is also emerging as one of the important domains in resilience research [4].

A number of conceptual and methodological issues arise in research into the role of religion in mental illness and health. The first of these is whether religion and spirituality should be regarded as synonymous. Many studies use measures that combine both into a single scale that does not distinguish one from the other [5,6] while others view them as two aspects of the same construct with separate subscales that will allow evaluation of each [7] or measure them as completely independent constructs [8]. This distinction is important since religion or spirituality may have similar or different roles in protecting against and/or modifying the impact of stressors on mental health and on other health-related outcomes. Many studies confound the two, notwithstanding the changing definition of spirituality in recent decades from one that was located within a religious framework to one that is secular [9]. In addition, some of the current definitions of spirituality are vague and conflate spirituality with happiness and general well-being [9], such as the frequently used World Health Organization Quality of Life Assessment—spiritual, religious and personal beliefs (WHOQOL– SRPB) questionnaire [6]. This can cause difficulties in understanding what the results mean.

The second consideration relates to whether the impact of religion/spirituality is on initial severity of depressive symptoms only or whether they also have an impact on response to treatment or recurrence. Cross-sectional studies suggest that attending regular church services is associated with a reduced risk of lifetime depressive illness, PTSD and alcohol use disorders and current suicidal ideation [10,11]. In a meta-analysis involving 147 studies (*n* = 98,975), Smith et al. [12] found a weak inverse relationship between religiousness and depression. King et al. [13] found that those with a spiritual outlook on life were at increased risk of depression compared to those who were engaged in religious practice. Abd Aleati et al. [14], in their systematic review of multiple faith groups, found that of the 74 studies identified, most described lower odds of depression, of anxiety disorders, of substance misuse problems, and of suicidal behaviours among those who had higher scores on religiousness or religious activity. This review identified studies conducted among a variety of age groups and among outpatients and inpatients, of which only two were longitudinal. Therefore, the question of causality cannot be answered [15] nor can the effects on outcome over time. 

Contrary to the above studies that have identified an association between church attendance and a reduced risk of psychiatric disorders, Leurent et al. [16], in their international study of primary care attenders, found that those with a religious or spiritual understanding of life had a higher incidence of depression than those with a secular life view. However, this finding varied by country and in particular those in the UK who had a spiritual understanding of life were the most vulnerable to the onset of major depression. Regardless of country, the stronger the spiritual or religious belief at baseline, the higher the risk of onset of depression. They found no evidence that spirituality protected against depression but they identified weak evidence that a religious view was possibly protective in two countries (Slovenia and the Netherlands). One possible explanation may be that depression reduces attendance at religious services and less spiritual or religious belief may be a consequence of depression rather than an aetiological factor. Li et al. [17], in their study of over 48,000 US nurses followed over 12 years, confirmed that religious service attendance reduced the risk of depression but also showed that those who were depressed were less likely to attend such services. These combined findings are of importance since they demonstrate a possible two-way relationship between depression and church attendance.

Longitudinal studies are required to answer questions regarding possible causal links between religions/spirituality and psychiatric disorders. These have examined whether religiousness and spirituality have similar or different effects on response to treatment and recurrence. Mihaljevic et al. [18] found that spirituality, not religiousness, was a significant predictor of recovery from depression among outpatients although his measure of spirituality was one which conflates spirituality with emotional wellbeing [6] rather than one which is focused on the supernatural. Na-Young et al. [19] also demonstrated that only spirituality was associated with a positive response to antidepressant treatment. Higher levels of spirituality were associated with a favourable response to treatment in depressed patients when baseline severity and treatment duration were controlled [19].

Miller et al. [20] found that those identifying a high personal importance of religion/spirituality had a 10% reduction in the likelihood of depression recurring among those already at high risk of major depression (by virtue of having a parent with major depression). Denominational affiliation or frequency of church attendance did not have any significant impact. On the other hand, Balbuena et al. [10], in a 14-year follow-up study, identified a lower risk of onset depression over time associated with, at least, monthly religious attendance but no association with spirituality. Rasic et al. [21] identified an interaction between gender and religious attendance on depression. Religious attendance at baseline lowered the odds of being depressed two years later among males who were depressed at the index assessment while females who attended church and were not depressed had lower odds of developing depression during the follow-up period. In other words, among men, religious attendance reduced the risk of chronicity in those depressed at baseline while, among women who were not depressed, it reduced the risk of becoming depressed.

A further consideration arises from the likely complexity of the relationship between religion/spirituality and mental illness. One question is whether religion/spirituality acts as a main effect or as a buffer. Most studies have focused on the role of religion as a main effect [11,22,23,24], but it might be that religion has a moderating (buffering) effect on symptom severity in certain groups. This was shown in several longitudinal studies [20,25,26], although Leurent et al. [16] found that religiousness or spirituality did not buffer the relationship between severe life events and onset of major depression. 

Above and beyond any impact of religiousness/spirituality on mental health, there is also the view that social support is important and that any effect of religion/spirituality comes from the support of others rather than religion/spirituality per se. Social support has been shown to be a consistent impact in determining severity of depressive symptoms [27,28], so it is of importance to examine its role in comparison to that of religion/spirituality on mental health as well. Some suggest that the impact of religion on depression is due to the social support that it provides rather than religious practice itself [29].

The present analysis was part of a larger study examining adjustment disorder and depressive episodes [30]. It was designed to overcome some of the methodological flaws found in other studies of religion and mental health mentioned above. The aim of the present analysis was to examine the role of religious practice (organised religious activity, non-organised religious activity) and personal religiousness (intrinsic religiousness) in determining the severity of depressive symptoms in the initial response to stressful life events and in relation to their six-month outcome. We specifically decided to omit measures of spirituality because of the problems in measuring this construct [9]. Based on the information from research to date, we examined which, if any, aspects of religiousness (organised religious activity, non-organised religious activity or personal religiousness) would have a main effect or would buffer the effects of life events on symptom severity in the cross-sectional study and on six-month symptom outcome. Furthermore, we wished to examine the role of social support, either as a main effect or as a buffer, in determining symptom severity at baseline and six-month outcome.

## 2. Materials and Methods 

### 2.1. Study Population

The methods of this study have been previously described in detail elsewhere [30] and are summarized briefly here. Participants were recruited from referrals to the liaison psychiatry services at three Dublin hospitals between May 2009 and June 2012. They were diagnosed clinically by the liaison psychiatrists with either a depressive episode (DE) or an adjustment disorder (AD) according to the ICD-10 classification [2]. They were also diagnosed by the research team using the Schedules for Clinical Assessment in Neuropsychiatry (SCAN) interview [31]. Patients were excluded if they had a substance abuse disorder, cognitive impairment, psychotic symptoms, were less than 18 years old, or unable to give informed consent to participation in the study. They also had to be competent in the English language. For the analysis in this study, we used the clinical diagnosis and the Beck Depression Inventory as a measure of symptom severity. *n* = 348 patients completed the first assessment of the study and *n* = 132 participants were followed up six months later.

### 2.2. Life Events

The List of Threatening Experiences (LTE) [32] was used to measure the number of life events that a participant experienced in the past two years. The response format is a simple yes or no answer to 12 possible life events. The LTE showed satisfactory results regarding retest reliability [33], concurrent validity [34], and sensitivity [33] in earlier studies.

### 2.3. Depressive Symptoms

Depressive symptomatology was measured with the revised Beck Depression Inventory (BDI-II) [35]. The 21 items are scored on a four-point Likert scale with higher scores indicating greater levels of depression. Results regarding the internal consistency, the convergent validity, and the factor structure were satisfactory in previous studies [36]. The internal consistency in the present study was Cronbach α = 0.90.

### 2.4. Social Support

The Oslo 3-items Social Support Scale (OSS) [37] measured ease in obtaining help from neighbours, the number of people to count on when serious personal problems arise, and perceived concern shown by others. A higher score is indicative of higher social support. The internal consistency in the present study was Cronbach α = 0.75.

### 2.5. Religiousness

Religiousness was assessed by the Duke University Religion Index (DUREL) [8]. The five item scale measures organised religious activity (ORA), non-organised religious activity (NORA), and intrinsic religiosity (IR). ORA consists of public religious activities, and NORA refers to religious activities performed in private such as praying, listening to religious programmes or else, while IR measures the degree of personal religious commitment or motivation. The response format of ORA and NORA is a 6-point Likert scale ranging from 1 (‘rarely/never’) to 6 (‘more than once a week (ORA) /day (NORA)’). The 5-point Likert scale for the three IR items ranges from 1 (‘definitely not true’) to 5 (‘definitely true’). Previous studies found promising results regarding factor structure [38] and convergent and discriminant validity [39]. The internal consistency in the present study was Cronbach α = 0.90. The authors caution against summation of the three subscales [8].

### 2.6. Data Analysis

The analyses were conducted using IBM SPSS statistics, version 23 [40]. We controlled for sex and age in all the analyses. No missing values were imputed prior to the analyses. There were 0.3% missing values on the life events measure and 2.3% on the depression measure. The outlier analysis using the Mahalanobis Distance [41] detected no multivariate outliers in the data. 

We performed hierarchical linear regression analysis to investigate the relationship between the predictor variables and depressive symptoms at t1. The independent variables entered into the model were sex, age (step 1), life events, social support, and religious activity (ORA/NORA/IR respectively) (step2). The interaction between life events and social support, and the interaction between life events and religious activity were entered as a third step to investigate buffering effects. Due to the restricted sample at t2, we used multiple linear regression analysis and no hierarchical approach for the longitudinal analysis. In case of significant interaction terms, we used a median split to differentiate between high and low scores on religious activity. Separated by low and high scores on religious activity, we performed a simple regression analysis with depressive symptoms as the outcome and life events as the predictor to receive beta-weights for the different groups. For both baseline and t2 data, we conducted separate analyses for the three aspects of religion as advised by the authors of the scale [8]. The total scores were standardised prior to analysis. All regression weights were estimated using bias-corrected and accelerated bootstrapping with 1000 bootstrap samples [42].

## 3. Results

The demographic characteristics of the sample can be found in Table 1. Women showed more intrinsic religiousness (IR) (t(344) = −2.415, *p* < 0.05) than men. The difference in the attendance of religious meetings was marginally significant (t(343) = −1.957, *p* = 0.051), with women, on average, attending organised religious meetings (ORA) more often than men. There was no difference in non-organised religious activity (NORA) in either sex. The correlations of study variables can be found in Appendix A Table A1.

Descriptive comparisons between the attrition and non-attrition subsample revealed that the attrition sample indicated fewer life events (attrition sample: M = 1.54, SD = 1.54; non-attrition sample: M = 2.01, SD = 1.83, t(239) = −2.44, *p* = 0.016) and higher social support (attrition sample: M = 10.26, SD = 2.45; non-attrition sample: M = 9.70, SD = 2.69, t(345) = 2.003, *p* = 0.046). There were no statistically significant differences in mean scores on the other relevant variables.

### 3.1. Hierarchical Regression for Depressive Symptoms at t1

Table 2 shows the results for the hierarchical regression with severity of depressive symptoms as the outcome. The independent variables entered into the model were sex, age, life events, social support, and religious activity (ORA/NORA/IR respectively). 

In the analysis including ORA, the R^2^ change for Model 3 was significant (F = 6.28, *p* < 0.05) and was therefore chosen as the interpretable model. The main effect of social support (*β* = −0.26, *p* < 0.001) and the interaction of life events x ORA (*β* = −0.19, *p* < 0.01) were significant. Further investigation of the interaction revealed that individuals with low ORA showed a stronger association between the number of life events and depressive symptoms at t1 (*β* = 0.30, *p* < 0.001) than individuals with high ORA (*β* = 0.04, n.s.), indicating a buffering effect of ORA on the relationship between life events and severity of depressive symptoms. 

In the analysis including NORA, the R^2^ change for Model 3 was not significant (F = 1.79, n.s.); thus, Model 2 was chosen as the interpretable model. The direct effect of life events (*β* = 0.11, *p* < 0.05) and social support (*β* = −0.27, *p* < 0.001) were significant but the direct effect of NORA was not (*β* = −0.02 n.s.).

In the analysis including IR, the R^2^ change for Model 3 was not significant (F = 2.50, n.s.); thus, Model 2 was chosen as the interpretable model. The direct effect of social support (*β* = −0.26, *p* < 0.001) was significant. The direct effect of life events (*β* = 0.10, *p* = 0.052) and IR (*β* = 0.11, *p* = 0.052) were marginally significant.

### 3.2. Multiple Linear Regression for Depressive Symptoms at t2

The results of the multiple linear regression in the longitudinal analysis can be found in Table 3. Analyses were conducted separately for ORA, NORA, and IR. In all three analyses, severity of depressive symptoms at t1 (all: *β* = 0.39, *p* < 0.001) was the strongest predictor of symptom severity at the six-month follow-up, and the effect of life events reported at t1 was also significant (ORA: *β* = −0.25, *p* < 0.01; NORA: *β* = −0.27, *p* < 0.01; IR: *β* = −0.26, *p* < 0.01). The effect of age was marginally significant in the analysis of ORA (*β* = 0.17, *p* = 0.52), and it was significant in the analysis of IR (*β* = 0.17, *p* < 0.05). 

We found a significant interaction effect for life events x NORA (*β* = −0.23, *p* < 0.01) and life events x IR (*β* = −0.15, *p* < 0.05), indicating a buffering effect of these on the relationship between life events and depressive symptoms at the six-month follow-up. Further investigation of the interaction effects revealed that individuals with low NORA showed a non-significant positive association between the number of life events and the severity of depressive symptoms at t2 (*β* = 0.14, n.s.), and individuals with high NORA showed a significant negative association between the number of life events and the severity of depressive symptoms at t2 (*β* = −0.42, *p* < 0.01). In other words, high levels of non-organised religious activity resulted in lower levels of depression in those with higher life event scores. The same pattern emerged for IR: The association between the number of life events and depressive symptoms at t2 was non-significant for individuals with low IR (*β* = 0.06, n.s.), and it was significantly negative for individuals with high IR (*β* = −0.38, *p* < 0.01).

## 4. Discussion

The present analysis examined the role of religiousness and social support in the initial and six-month outcome of those with depressive symptoms in DE and AD in response to stressful life events. It is one of the few studies in this field to use a longitudinal design, thus contributing to our understanding of the likely causal role of religiousness in mental health and in determining the outcome of ill health. Several aspects of religious activity were measured as there is evidence of differential effects on depressive symptoms [12]. The three domains of religious activity measured by the DUREL [8] were analysed separately to establish whether they evidenced main effects or a buffering role.

In the first analysis of data at t1, ORA buffered the impact of life events on depression severity at baseline. In the second analysis, more life events and less social support were associated with higher depressive symptomatology at baseline while NORA showed no association with outcome. In the third analysis, higher depressive symptomatology was associated with more life events, less social support, and less IR. So, neither NORA nor IR showed a buffering effect of life events on the overall depression score. For follow-up analysis at six months, NORA and IR did buffer the impact of life events on the depressive symptom score, and the best predictors were severity of depressive symptoms at t1 and the number of life events at t1. The role of age was variable in its association and social support did not contribute to the severity of depression at six months. The finding that the number of life events was positively associated with initial depression scores and with the outcome at six months is in line with earlier research on the role of critical life events in symptom development [1].

The results in the present study showing that ORA has an influence on depressive symptoms is in line with other studies indicating the role of church attendance and other organised activities in reducing the risk of depression [10,14] and other disorders [11] with one pointing to a 22% reduction in risk of depression [10]. The finding of a buffering effect between ORA and life events on severity of depressive symptomatology is in line with the meta-analysis by Smith et al. [12] and with a study by Kasen et al. [25]; the latter showed a decreased risk for developing a mood disorder in individuals at high risk who attended church more regularly. 

It was surprising that NORA had no impact on index symptom severity since the benefits of such private activities as prayer have been identified in other studies, specifically on severity of depression [43,44] and on coping with depression [45]. The reasons for this discrepancy are unclear but may be due to methodological issues since Wachholtz [45] and Ronneberg [44] were both ecological and so had larger samples than this study. However, they used ecological and not clinically derived data, potentially leading to different findings. It is also possible that religious cultural differences between the countries in which these various studies were carried out may have played a role.

The third component, intrinsic religiousness (IR), was associated with fewer depressive symptoms. Smith et al. [12] found that the type of religiousness, i.e., intrinsic (valuing religiousness as a means of getting close to God) versus extrinsic (for status it confers), had different effects with the intrinsic approach reducing and the extrinsic increasing depressive symptoms. Our findings are in line with the attenuating effect of IR. However, they seem to contradict the findings of Balbuena et al. [10] who found that neither the importance of spiritual values nor identifying as a spiritual person was protective against depression. It is possible that the variable we measured, i.e., personal importance of religiousness, differed from that being measured in the spirituality dimension of Balbuena et al. [10].

For the six-month follow-up data, our study showed that NORA and IR buffered the effects of life events on symptom severity as an outcome. This is broadly in line with previous longitudinal studies [44,46] showing that non-organised religious activities such as prayer etc. impacted positively on the outcome for those who were depressed at baseline, although this was examined as a main effect only in these studies. The buffering impact of IR on symptom severity at follow-up in our study replicates the findings of Miller et al. [20] that high personal commitment to religion buffered the risk of recurrence in those who were at high risk, having had a prior episode.

Social support was included in all analyses to control for its effects on outcome and to examine whether the benefits of religiousness were due to the social support that organised religious activity is associated with as claimed by some [29]. We found a main effect of social support only on depressive symptomatology at t1 with higher social support being associated with fewer symptoms, but it did not buffer the effect of life events either at initial assessment or longitudinally. Our results show that the benefit of religious activity is not just simply a product of the social support that it offers but something over and above this [47].

With regard to the results overall, they show that church attendance is important in moderating depression severity when the individual is exposed to stressful life events. On the other hand, the personal belief system (IR and NORA) seems to be more relevant to the decrease of depressive symptoms over time than is church attendance. Social support only contributed as a main effect to the initial severity but unlike the various dimensions of religiousness, it did not buffer the impact of life events at any of the time points examined in the study.

It is difficult to interpret the relevance of the differential effects of the various components of religiousness at the time of initial assessment and when measured after a certain period. The impact of organised religious practice in moderating the effect of life stressors on mood may be due to the reappraisal of stressful events that religious practice facilitates such as the belief that events happen for a reason, that events can lead to spiritual growth, and the unique support that a regular faith-based community can offer. However, this may also be an indicator of reverse causation due to the impact of depression in reducing religious attendance. A bidirectional effect has been observed in the largest study to date with depressive symptoms predicting a 26% reduction in religious attendance and religious attendance predicting a 29% reduction in depressive symptoms [17].

The results of our study indicate that the effects of non-organised religious practice such as prayer, scripture readings etc., and intrinsically derived religious faith take time to impact upon stressor-induced depressive symptoms. Perhaps the reflective aspects that non-organised religious activity stimulates require a more deliberative and reflective approach than does the style of church worship. Attributes like hope and meaning take time to distill cognitively and thus their impact may not be present immediately but only emerge over time. Others [48] have noted that the association of religiousness and fewer depressive symptoms appeared to be particularly strong when religiousness was measured in terms of public religious involvement or intrinsic religious motivation and less so when religiousness was measured in terms of private religiousness. The explanations for the differential impact of the various religious dimensions require further study in clinical populations. This will be challenging since the measures of the various religious constructs and their association with symptoms may be highly inter-correlated. They may also be tapping into higher order religious attributes that are difficult to measure using questionnaires, instead requiring imaging techniques such as those used by others [49]. 

Regarding the implications of these findings in clinical practice, they may be of assistance to mental health practitioners, pastoral counsellors, and chaplains when dealing with people of faith. Learning to discuss and then harness religious practices is regarded as a component of resilience and these findings suggest that, for specific groups, encouragement around these activities is likely to be beneficial. There may be some people with mental health problems who have drifted from religion-related activities, not through loss of faith per se but because of their illness or pressure from competing demands, but still accept that religious practice and faith have benefits. These might sensitively be encouraged to reengage. There is a danger that over-zealous practitioners might try to advocate these practices to non-believers and this would be unacceptable. Practitioners should adhere to the guidelines published by the regulatory bodies regarding practices and interventions of this nature.

The present results should be interpreted with several limitations in mind. First, the data stem from a very specific sample of patients of the liaison psychiatry services in an urban area. This limits the generalisability of the results to all patients with depressive episodes or adjustment disorder in the general population and to those occurring in other cultures. On the other hand, this study did use a clinical sample, unlike many studies in this field which used ecological data on subjects who were only screened and are likely to differ substantially from patient samples such as ours. Second, the sample size for the longitudinal analysis was rather small and due to power considerations, we applied a more conservative method of analysis. The longitudinal sample differed from the cross-sectional sample and reported more life events experienced and less social support. The significant effects of life events on longitudinal outcome should therefore be interpreted with caution since these buffering effects might only be evident in individuals who experienced more life events. Third, the independent variables were all assessed, initially, during a current episode of depressive illness or adjustment disorder. Hence, we were not able to entirely separate cause and effect in the initial response to the questionnaire, e.g., depressive symptoms could also reduce church attendance as shown by [17]. Further studies, in different populations and cultures, should be conducted with larger samples, using prospective designs. These should also consider other variables that might impact on outcome, such as personality and treatments. In future studies, a systematic assessment of the nature and amount of treatment individuals received could help to identify the specific role that religious activities have in treating depressive illness, alone or in conjunction with pharmacological and/or psychological treatments, as has been identified in older people with depressive illness [50].

## 5. Conclusions

Notwithstanding the limitations, this study is important in that it has provided information on the differential effect of various dimensions of religiousness not often studied. In addition, by examining the contribution of these dimensions both as main effects and as buffers and the respective roles of social support, various aspects of religiousness have been clarified. Moreover, this analysis was not post-hoc but was included in the original proposal. Finally, the longitudinal design has shed some light on the possibility of a causal link between religiousness, severity of depressive symptoms and outcome.

## Figures and Tables

**Table 1:** Demographic characteristics of the sample.

Variables	*M* (*SD*)			Statistic for Gender Comparison	
	Full Sample	Male	Female	*t* (*df*)	*p*
Age	43.51 (14.32)	43.72 (14.97)	43.40 (13.99)	0.199 (343)	0.842
Depressive symptoms t1	28.11 (11.78)	28.41 (11.72)	27.95 (11.83)	0.343 (338)	0.732
Depressive symptoms t2	14.11 (12.50)	16.35 (14.41)	12.92 (11.26)	1.509 (130)	0.134
Life events	1.72 (1.67)	1.68 (1.67)	1.74 (1.67)	−0.306 (345)	0.760
Social Support	10.05 (2.55)	9.83 (2.56)	10.17 (2.55)	1.179 (345)	0.239
Organised religious activities (ORA)	2.01 (1.60)	1.78 (1.62)	2.13 (1.58)	−1.957 (343)	0.051
Non-organised religious activities (NORA)	1.59 (1.83)	1.52 (1.90)	1.63 (1.79)	−0.571 (344)	0.569
Intrinsic religiousness (IR)	6.25 (6.00)	5.58 (4.04)	6.62 (3.68)	−2.415 (344)	0.016

Note. *M* = mean; *SD* = standard deviation; *t* = value of t-distribution; *df* = degrees of freedom; *p* = statistical significance; t1 sample: *n* = 348, *n* = 122 male, *n* = 226 female.

**Table 2:** Hierarchical linear regression for depressive symptoms—divided by ORA, NORA, IR (standardized coefficients; *n* = 348).

Predictors	Model 1			Model 2			Model 3		
	*β (95% Bca CI)*	*SE β*	*p*	*β (95% Bca CI)*	*SE β*	*p*	*β (95% Bca CI)*	*SE β*	*p*
*Moderator: ORA*									
Sex	−0.05 (−0.27; 0.18)	0.11	0.683	0.02 (−0.20; 0.24)	0.11	0.838	0.02 (−0.19; 0.24)	0.11	0.862
Age	−0.10 (−0.19; 0.01)	0.05	0.074	−0.03 (−0.12; 0.07)	0.06	0.638	−0.02 (−0.11; 0.08)	0.06	0.757
Life Events				0.10 (−0.02; 0.21)	0.05	0.065	0.07 (−0.04; 0.17)	0.06	0.217
Social Support				−0.27 (−0.38; -0.15)	0.05	0.000	−0.26 (−0.37; −0.15)	0.05	0.000
ORA				−0.14 (−0.24; -0.19)	0.06	0.018	−0.14 (−0.24; −0.02)	0.06	0.013
Life Events x Social Support							0.00 (−0.09; 0.08)	0.05	0.971
Life Events x ORA							−0.19 (−0.29; −0.10)	0.05	0.001
*R* ^2^	0.00			0.12			0.14		
*F* for change in *R*^2^	1.68			15.03 ***			6.28 **		
*Moderator: NORA*									
Sex	−0.05 (−0.27; 0.17)	0.11	0.653	−0.01 (−0.22; 0.20)	0.11	0.907	−0.02 (−0.22; 0.21)	0.11	0.892
Age	−0.10 (−0.20; 0.01)	0.05	0.076	−0.07 (−0.18; 0.04)	0.06	0.209	−0.07 (−0.17; 0.05)	0.06	0.222
Life Events				0.11 (0.00; 0.21)	0.05	0.048	0.08 (−0.04; 0.20)	0.06	0.156
Social Support				−0.27 (−0.39; -0.18)	0.05	0.000	−0.27 (−0.38; −0.18)	0.05	0.000
NORA				−0.02 (−0.14; 0.09)	0.06	0.728	−0.03 (−0.15; 0.08)	0.06	0.619
Life Events x Social Support							−0.03 (−0.13; 0.05)	0.05	0.462
Life Events x NORA							−0.10 (−0.21; 0.00)	0.03	0.083
*R* ^2^	0.00			0.10			0.11		
*F* for change in *R*^2^	1.68			12.94 ***			1.79		
*Moderator: IR*									
Sex	−0.05 (−0.28; 0.16)	0.11	0.653	0.01 (−0.21; 0.26)	0.11	0.910	0.01 (−0.21; 0.25)	0.11	0.926
Age	−0.10 (−0.20; 0.00)	0.05	0.076	−0.04 (−0.15; 0.07)	0.05	0.438	−0.05 (−0.15; 0.07)	0.05	0.398
Life Events				0.10 (0.00; 0.20)	0.05	0.052	0.09 (−0.04; 0.20)	0.06	0.136
Social Support				−0.26 (−0.38; -0.16)	0.05	0.000	−0.26 (−0.37; −0.15)	0.05	0.000
IR				−0.11 (−0.22; 0.00)	0.06	0.052	−0.11 (−0.21; −0.01)	0.06	0.054
Life Events x Social Support							−0.03 (−0.14; 0.07)	0.05	0.533
Life Events x IR							−0.11 (−0.22; −0.01)	0.05	0.039
*R* ^2^	0.00			0.11			0.12		
*F* for change in *R*^2^	1.68			14.31 ***			2.50		

Note. *β* = beta-weight; *Bca CI* = bias-corrected and accelerated bootstrapped confidence interval; *SE* = standard error; *p* = statistical significance; ORA = organised religious activities; NORA = non-organised religious activities; IR = intrinsic religiousness; *R^2^* = variance explained; *F* = value from F-distribution. * *p* < 0.05; ** *p* < 0.01; *** *p* < 0.001.

**Table 3:** Multiple linear regression for depressive symptoms at t2—divided by ORA, NORA, IR (*n* = 132).

Predictors	*β (95% Bca CI)*	*SE β*	*p*
*Moderator: ORA*			
Sex	−0.28 (−0.56; 0.03)	0.16	0.107
Age	0.17 (0.00; 0.36)	0.09	0.052
Depressive Symptoms t1	0.39 (0.23; 0.57)	0.08	0.000
Life Events t1	−0.25 (−0.41; −0.10)	0.08	0.002
Social Support t1	0.00 (−0.19; 0.17)	0.08	0.972
ORA t1	0.00 (−0.17; 0.16)	0.08	0.991
Life Events x Social Support	−0.01 (−0.12; −0.10)	0.06	0.916
Life Events x ORA	−0.10 (−0.28; 0.05)	0.07	0.171
*R* ^2^	0.25		
*Moderator: NORA*			
Sex	−0.27 (−0.59; 0.02)	0.16	0.082
Age	0.14 (−0.03; 0.30)	0.08	0.090
Depressive Symptoms t1	0.39 (0.22; 0.56)	0.07	0.000
Life Events t1	−0.27 (−0.41; −0.12)	0.08	0.001
Social Support t1	−0.01 (−0.17; 0.15)	0.08	0.939
NORA t1	0.02 (−0.14; 0.17)	0.07	0.765
Life Events x Social Support	−0.06 (−0.19; 0.04)	0.06	0.339
Life Events x NORA	−0.23 (−0.36; −0.13)	0.07	0.001
*R* ^2^	0.30		
*Moderator: IR*			
Sex	−0.25 (−0.56; 0.02)	0.16	0.109
Age	0.17 (−0.01; 0.37)	0.08	0.038
Depressive Symptoms t1	0.39 (0.23; 0.54)	0.07	0.000
Life Events t1	−0.26 (−0.43; −0.11)	0.08	0.001
Social Support t1	−0.01 (−0.16; 0.14)	0.08	0.946
IR t1	−0.05 (−0.22; 0.10)	0.08	0.459
Life Events x Social Support	−0.02 (−0.14; 0.08)	0.06	0.739
Life Events x IR	−0.15 (−0.32; −0.01)	0.07	0.033
*R* ^2^	0.27		

Note. *β* = beta-weight; *Bca CI* = bias-corrected and accelerated bootstrapped confidence interval; *SE* = standard error; *p* = statistical significance; ORA = organised religious activities; NORA = non-organised religious activities; IR = intrinsic religiousness; *R^2^* = variance explained; *F* = value from F-distribution.

## Appendix A

**Table A1:** Partial correlations between study variables (controlled for sex and age).

Variables	2.	3.	4.	5.	6.	7.
1. Depressive Symptoms	0.429 ***	0.175 ***	−0.310 ***	−0.184 **	−0.079	−0.169 **
2. Depressive Symptoms t2	1	−0.188 *	−0.117	−0.083	0.026	−0.139
3. Life Events		1	−0.239 ***	−0.109 *	−0.113 *	−0.102
4. Social Support			1	0.113 *	0.084	0.138 *
5. Organised religious activities (ORA)				1	0.571 ***	0.674 ***
6.Non-organised religious activities (NORA)					1	0.597 ***
7. Intrinsic religiousness (IR)						1

Note. t1 sample: *n* = 348; t2 sample: *n* = 132. * *p* < 0.05 ** *p* < 0.01 *** *p* < 0.001.
